# Microalgae enhanced plant growth and yield, and improved leaf color in soilless lettuce

**DOI:** 10.1038/s41598-025-24004-9

**Published:** 2025-11-17

**Authors:** İbrahim Memeli, Yüksel Tüzel, Önder Uysal, Tunç Durdu, Fatmagül Özge Uysal, Omar Saley Harouna, Kamil Ekinci, Nazim S. Gruda

**Affiliations:** 1 Faculty of Agriculture, Department of Horticulture, Ege University, İzmir, 35100 Turkey; 2 Department of Agricultural Machinery and Technology Engineering, Isparta University of Applied Sciences, Isparta, 32260 Turkey; 3 Teknotar Ltd. Co, İzmir, 35070 Turkey; 4 Department of Horticultural Sciences, University of Bonn, INRES – Institute of Crop Science and Resource Conservation, 53113 Bonn, Germany

**Keywords:** *Lactuca sativa*, *Chlorella* sp., *Scenedesmus obliquus*, Soilless culture, Heatmap, Plant physiology, Ecology

## Abstract

Microalgae can stimulate plant growth through bioactive compounds such as cytokinins, gibberellins, and abscisic acid. Here, we present novel insights into applying live microalgae cultures—*Chlorella* sp. and *Scenedesmus obliquus*—at different concentrations in lettuce cultivation, cv. ‘Emocion’. Plants were grown in perlite under greenhouse conditions. Microalgae suspensions were prepared using fresh weight concentrations of 0.775, 0.0775, and 0.00775 g L^−1^, corresponding to cell densities of 2 × 10^7^, 2 × 10^6^, and 10^5^ cells mL^−1^, respectively, and applied to the substrate as 140 mL per plant. The control received no microalgae application. *Chlorella* sp. significantly improved head weight and total yield, with the 0.775 g L^−1^ concentration being the most effective. Total yield increase was 18.3% compared to the control. For *S. obliquus*, the optimal effect was observed at 0.0775 g L^−1^, yielding a 2.7% higher yield than the control. Heatmap analysis revealed two primary treatment clusters, separating *Chlorella* sp. from *S. obliquus* and the control, with *Chlorella* sp. closely associated with yield-enhancing parameters. We concluded that *Chlorella* sp. application can effectively increase yield with 0.775 g L^−1^ identified as the most beneficial dose. Future research should investigate the mechanisms of microalgae–plant interactions and evaluate their applicability across various crops and cultivation systems.

## Introduction

Any substance or microorganism applied to plants to enhance nutritional efficiency, abiotic stress tolerance, and/or crop quality traits, regardless of nutrient content, is a plant stimulant^1^. According to EU Regulation 2019/1009 of the European Parliament and of the Council, “*certain* *substances*,* mixtures and micro-organisms*,* referred to as plant biostimulants*,* are not as such inputs of nutrients*,* but nevertheless stimulate plants’ natural nutrition processes*”^2^.

In the past fifty years, microorganisms have been successfully applied across numerous fields, including medical technology, human and animal health, food processing and safety, genetic engineering, environmental protection, and agricultural biotechnology, with notable success in converting agricultural and household waste. Advances in technology have enabled the commercial development of a diverse range of microbial cultures and inoculants for treating domestic waste.

Microalgae are recognized as environmentally friendly biostimulants/biofertilizers that increase crop yield and quality^3^. In search of more sustainable and environmentally-friendly solutions to increase agricultural productivity, researchers have focused their attention on bio-based products, with microalgae and cyanobacteria emerging as a valuable resource for crop production and conservation due to their potential for biofertilization and biostimulants^4^. Planktonic organisms, a group of organisms adapted to living suspended in seas and inland waters such as lakes, reservoirs, and rivers, are organisms that cannot move on their own, but can be displaced by water movements (winds, currents, tides)^5^. Phytoplankton species, which constitute the plant members of planktonic forms, are found singly or in colonies^6^. The most important organic matter producers in aquatic habitats are phytoplankton. These microscopic organisms are classified according to their size as picoplankton, less than 2 μm, nanoplankton, between 2 and 20 μm, and microplankton, up to 200 μm ^7^. Because they contain chlorophyll and require sunlight for photosynthesis, most phytoplankton are buoyant and float in the upper part of the water, where sunlight penetrates the water^8^. In addition to light and oxygen (O_2_), they require basic, simple inorganic chemical nutrients such as phosphate (PO_4_) and nitrate (NO_3_), as well as carbon in the form of carbon dioxide (CO_2_). Their impact on humankind includes their role in controlling atmospheric carbon dioxide, converting inorganic nutrients such as phosphate, nitrate, and carbon dioxide into larger and more complex organic molecules essential for life, and detecting the biotoxins they produce^9^.

Phytoplankton are a wide variety of unicellular autotrophs that can be considered a good source of nutrients. The elemental composition of their cells includes primary macronutrients, such as nitrogen (N), potassium (K), phosphorus (P); secondary macronutrients, such as calcium, magnesium, and sulfur; and micronutrients, such as iron, manganese, zinc, and copper^10^. The use of phytoplanktonic algae in agriculture dates back thousands of years. There have been observations of positive effects of using algae collected from the coasts directly or after composting^3^. In the 20th century, products derived from macro and micro algae extracts attracted the attention of farmers worldwide^11^. A wide range of biologically active compounds from algae has also been shown to have promising effects in crop production^12^. Metabolites in microalgae have been reported to increase soil fertility, confer resistance to abiotic stress to plants, stimulate defense response against pathogens and infection, and improve the uptake of nutrients such as N, P, and K from soil^3,13,14^. In recent years, many studies have reported the use of microalgae to enhance the yield and quality of various crops^15–19^.

Microalgae species have been utilized for multiple purposes. *Chlorella vulgaris* has been employed to remove nitrate, sulfate, and phosphate from wastewater^20^. It also plays a role in conserving nutrients in soilless cultivation^21^ and treating wastewater in aquaculture^22,23^. Moreover, *C. vulgaris* is used to purify water in nurseries^24^. Its extracts have been effective in germinating seeds^25^ and are beneficial for eco-hydroponic cultivation^26^. Additionally, it is studied for its effects on plant growth^27^. *Scenedesmus obliquus* extract and its fractions are rich in antimicrobial and anticancer compounds^28^ and used for wastewater treatment^29,30^.

Sustainable and cost-effective tools/strategies are required to reduce the environmental impact of agricultural practices. Soilless culture techniques present an innovative and resource-efficient pathway for sustainable horticultural production. Moving horticultural production from open fields to greenhouses allows all environmental conditions to be controlled better. Soilless culture additionally controls rootzone conditions, delivering precise nutrients to roots, thus enabling better growing conditions and higher yields^31^. Soilless culture systems refer to all plant cultivation methods without using natural soil, classified into liquid-based and solid media systems. Among liquid-based approaches, hydroponics is most common, where plant roots grow directly in nutrient solutions supplying water and essential minerals. Solid media systems use substrates providing physical support for delivering water and nutrients^32–34^. Lettuce benefits from hydroponic systems, especially floating systems, which are water-efficient and reuse large water volumes for fertilization, oxygenation, and temperature control, sometimes with a minimum of substrate used^35,36^. This closed system offers security, enables multiple crop cycles annually, and maximizes nutrient use with minimal waste. It requires little space, labor, and energy, making it an environmentally friendly and cost-effective method^18,37^. Aquaponic systems integrate fish farming with plant cultivation, where fish waste provides nutrients for plants while plants filter water for fish^38^. Also, substrate culture is used to cultivate lettuce. These soilless systems have gained widespread adoption globally, particularly in regions with limited arable land or adverse climatic conditions, offering year-round production and reduced water consumption compared to conventional agriculture^32,39^. The integration of microalgae into these systems as biostimulants represents a sustainable approach aligned with circular bioeconomy principles. The integration of microalgae into hydroponic systems as biostimulants represents a sustainable and eco-friendly approach aligned with circular bioeconomy principles^40^.

Despite these advances and earlier studies on microalgae extracts in agriculture, the direct application of live microalgae cultures in soilless systems is poorly understood, especially concerning species such as *C. vulgaris* and *S. obliquus*. This represents a significant knowledge gap regarding the intricate interactions between plant roots and actively growing microalgae and the optimal application strategies for different live microalgae species in these controlled environments.

To address this critical gap and offer insights for sustainable agriculture, here, we examined the effects of introducing live freshwater microalgae (*S. obliquus* and *Chlorella* sp.) into a substrate culture on the growth and yield parameters of lettuce (*Lactuca sativa* L.). We conducted experiments with different concentrations to ascertain (1) whether live algae improve growth in comparison to controls, (2) which species performs better, and (3) find the ideal dosage for lettuce production. We hypothesized that the direct application of live microalgae cultures would significantly improve lettuce growth, yield, and leaf color in a soilless perlite system compared to a control without microalgae. Furthermore, we hypothesized that *Chlorella* sp. and *S. obliquus* would exhibit differential efficacies in enhancing lettuce growth and yield, and that a distinct optimal concentration would be determined for each microalga to maximize specific production parameters, such as head weight and total biomass.

## Materials and methods

The study was conducted in an unheated greenhouse (38º27′16.20″N, 27º13′17.50″E) with polyethylene cover belonging to Ege University Faculty of Agriculture, Department of Horticulture (Bornova/Izmir, Türkiye).

### Plant material

In the present study, we used the plant material of curly salad (*Lactuca sativa* L. var. crispa) variety ‘Emocion’ (Rijk Zwann). Seedlings were obtained from Sancak Fide (İzmir).

### Microalgae used and their properties

The microalgae used in this research, *Chlorella* sp. and *S. obliquus*, were obtained from the Microalgal Biomass Laboratory of Isparta University of Applied Sciences, Department of Agricultural Machinery and Technology Engineering.

**Fig. 1:**
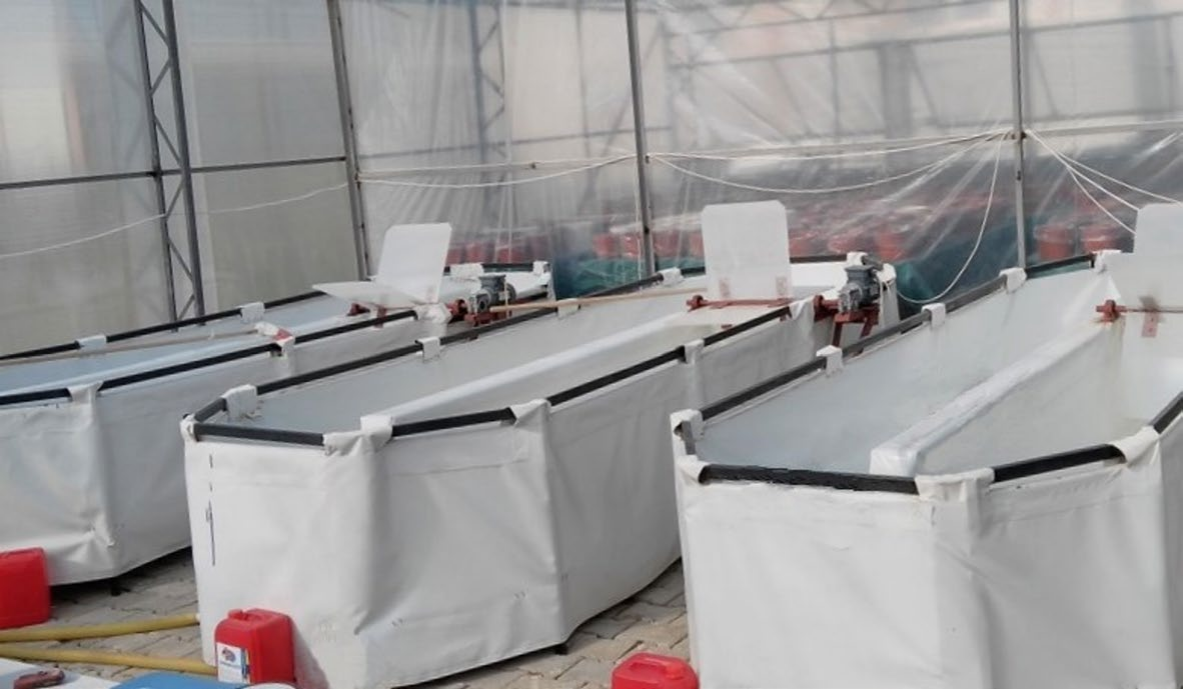
Raceway systems for Cultivation of *Chlorella* sp. and *S. obliquus*.

*Chlorella* sp. (SAG 242.80) and *S. obliquus* (ACUF 342) were grown in 250 ml Erlenmeyer flasks at the first stage, then transferred to 2000 ml Erlenmeyer flasks to increase the density of microalgae. Here, the pH of both microalgae strains was maintained at 7.0. After reaching sufficient density, the microalgae strains were cultivated in two one-ton capacity channel-type ponds installed in the greenhouse (Fig. 1). The basal medium prepared according to Uysal and Ekinci^41^ was used as the nutrient medium for both strains. Basal medium (ES “Erddekokt + Salze”) consisted of macronutrients, a soil extract, and a micronutrient solution. Per liter of medium, 20 mL of 1 g 100 mL^−1^ KNO_2_, 20 mL of 0.1 g 100 mL^−1^ K_2_HPO_4_, and 20 mL of 0.1 g 100 mL^−1^ MgSO_4_·7 H_2_O were added, along with 30 mL of soil extract and 5 mL of micronutrient solution. The final volume was completed to 1 L with de-ionized or distilled water. The micronutrient solution was prepared by mixing appropriate volumes of ZnSO_4_·7 H_2_O, MnSO_4_·4 H_2_O, H_2_BO_2_, Co(NO_2_)_2_·6 H_2_O, Na_2_MO_4_·2 H_2_O, and CuSO_4_·5 H_2_O in distilled water, with the addition of 0.7 g FeSO_4_·7 H_2_O and 8 g EDTA (Triplex III, Merck). Microalgae were harvested from the two ponds after reaching sufficient density. Dry biomass was determined using Whatman GF C^−1^ filter papers with a pore diameter of 0.45 μm using a precision balance with 0.0001 g sensitivity. One mL of culture was filtered using sample filter papers, which were then dried in an oven at 105 °C for 4 h. The dried filter papers were weighted using a precision balance and the dry weight per liter was calculated in grams (g)^41^.The properties of the harvested biomass of *Chlorella* sp. and *S. obliquus* are given in Table 1.

**Table 1:** The properties of the harvested biomass of *Chlorella* sp. and *S. obliquus*.

	*S. obliquus*	*Chlorella* sp
pH	9.2	9.3
N (%)	5.93	5.55
P (%)	1.02	1.07
K (%)	0.99	1.36
Ca (%)	10.14	6.5
Mg (%)	1.37	1.12
Fe (ppm)	2201.75	1304.01
Cu (ppm)	14.16	48.44
Mn (ppm)	1821.12	31.43
Zn (ppm)	653.32	44.34

Total nitrogen (TN) of the microalgae biomass were analyzed using CN analyzer (Vario MACRO CN Elemental analyzer). Dried microalgae were wet digested to analyze nutrients (P, K, Ca, Mg, Fe, Cu, Mn, and Zn) using microwave digesting system. Phosphorous (P) was determined using spectrophotometer, and the other nutrients were measured with atomic absorption spectrophotometer^42^.

To obtain a biostimulant/biofertilizer from microalgae cultivation, cells were harvested at the transition from exponential to stationary growth phase. At this stage, growth ceased due to nitrogen and phosphate depletion in the basal medium and deficiencies in potassium, magnesium, sulfate, and trace elements. Harvested microalgae were stored at + 4 °C for less than three months. Since nitrogen, phosphate, and potassium were largely consumed by the stationary phase, the stored medium was expected to retain magnesium, sulfate, and unconsumed trace elements. Cai et al. ^43^ demonstrated that magnesium sulfate (MgSO_4_·7 H_2_O) application enhanced growth parameters, yield, and root quality. The remaining micronutrients in the medium were anticipated to provide enrichment effects for the microalgae-based biostimulant/biofertilizer through their positive influence on enzyme activities, quality, stress tolerance, and reproductive processes^44^. Microalgae were harvested directly without washing and stored in liquid form under appropriate conditions. After harvest, microalgae were stored in the dark at + 4 °C for use within 3 months. Cell viability under these conditions was monitored using a Neubauer hemocytometer and microscopic counting with Trypan Blue.

Live microalgae were used in this study for their intended role as biostimulants and biofertilizers. To maintain viability, the cultures were stored in a cool, dark environment until application, thereby preventing spoilage. The underlying principle is that living cells can actively interact with the substrate and potentially modify their own metabolism, while over time, cell degradation may also release bioactive compounds that influence lettuce growth. These aspects of soilless cultivation and microorganism use are now elaborated in the Introduction and Methods sections.

### Production system

Substrate culture was used in the study. For this purpose, 25-liter plastic horizontal pots (S334 Model, Ceren Plastik, Yenişehir/İzmir) with dimensions of 75 × 23 × 16 cm were used. Perlite, an aluminum silicate of volcanic origin heated to 1000 °C, was used as an inorganic growing medium^45^. Some physical and chemical properties of perlite is given in Table 2. A drip irrigation system consists of a main tank, a pump, a booster, a pressure regulator, a disk filter, a main pipeline, water meters, solenoid valves, a lateral pipeline, and drippers was used. One lateral pipeline was installed in each row of the pots, and an arrangement was made to have one dripper for each plant. Irrigation water was applied with an automation system. The first irrigation was made at 10:00 am, and was carried out four times a day for 3 min at 2-hour intervals with a leaching rate of 25–30%.

The complete nutrient solution was applied to the plants via a drip irrigation system two days after planting. The chemical composition of nutrient solution was (mg L^−1^): N 150, P 50, K 150, Ca 150, Mg 50, Fe 5, Mn 0.5, B 0.5, Zn 0.05, Cu 0.03 and Mo 0.02 ^46^. The pH of the prepared nutrient solution was kept at 5.5–6.5, and the electrical conductivity (EC) was kept between 1.8 and 2.2. Fertilizers were prepared as a stock solution in separate tank of 150 L volume and microelement solutions in 10 L. Then, they added the water in calculated amounts in the main tank. The nutrient solution was applied to the root zone using an open drip irrigation system. The drained solution was collected in the relevant tanks, and the pH and EC of the samples were measured.

**Table 2:** Some properties of perlite.

	Value	Unit
pH	8.29	
EC	0.09	mS cm^− 1^
Total salt	0.0029	%
Saturation	440	
Organic matter	0.03	%
Total N	0.00	%
P	1.70	ppm
K	31.12	ppm
Ca	282.70	ppm
Mg	12.55	ppm
Fe	5.6	ppm
Cu	0.2	ppm
Zn	1.1	ppm
B	0.0	ppm
Mn	0.6	ppm

The average temperatures inside the greenhouse ranged between 2.86 °C and 38.54 °C. The mean temperatures were 16.15, 13.92, 14, and 12.81 °C in November, December, January, and February, while average relative humidity was 73.18, 79.78, 84.79, and 59.1%. Solar radiation (Rs) varied between 10.26 and 110.94 MJ m^−2^ day^−1^ throughout production.

### Setting up the trial

**Fig. 2:**
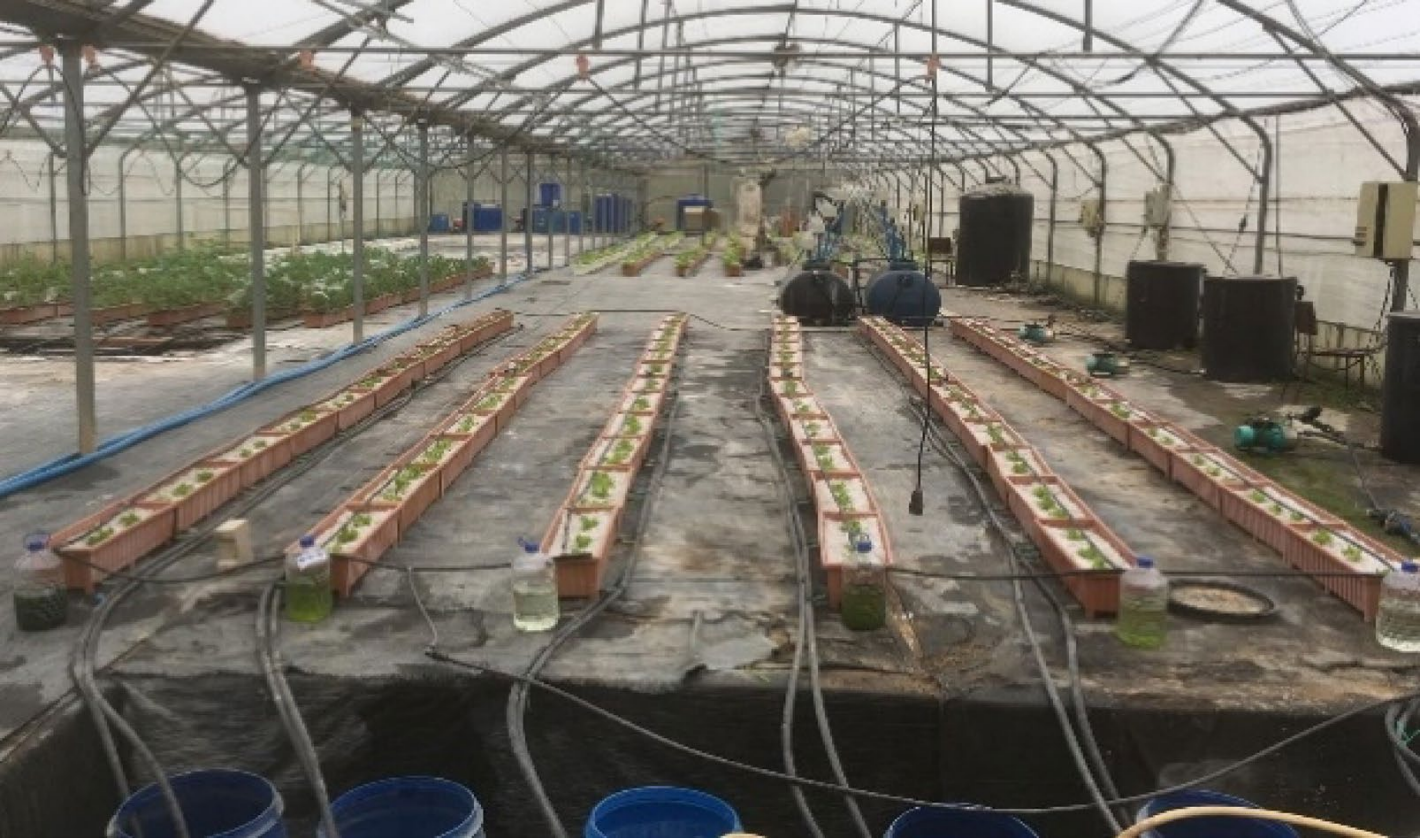
A view of trial after planting.

The experiment was conducted according to the randomized block design with 3 replicates (Fig. 2). Each plot had six pots and three plants (3.63 plants m^−2^) in each pot. For the plant trials in the study, three distinct biomass densities from both strains were maintained at + 4 °C for the biofertilizer trial (Table 3). The initial concentrations of the microalgae suspensions were 0.775, 0.0775, and 0.00775 g fresh weight L^−1^, corresponding to cell concentrations of 2 × 10^7^, 2 × 10^6^, and 2 × 10^5^ cells mL^−1^ for both microalgae species. Microalgae were applied at 140 mL per seedling. Cell concentrations were determined by microscopic counting using a Neubauer Hemocytometer. Plants without the microalgae treatment constituted the control group. A total of two treatments were performed on the 22nd and 29th of November 2019.

**Table 3:** Trial topics and intensities.

Microalgae	Dilution factor	Density (g L^−1^)	Application abbreviation name
*Chlorella* sp.	1	0.775	Cs(1)
	10	0.0775	Cs(10)
	100	0.00775	Cs(100)
S. obliquus	1	0.775	So(1)
	10	0.0775	So(10)
	100	0.00775	So(100)

### Measurements made

#### Measurements related to plant growth

The plants that reached harvest maturity were harvested early in the morning, and measurements and observations were made to determine the effects of the microalgae used in the study on the plants. Plant height (cm): After harvesting, six plants selected from each subject were measured from the beginning of the root to the tip of the plant with the help of a meter. Plant diameter (cm): The circumference of the harvested plants was measured from the widest part of the circumference with the help of a ruler on one axis. Root collar diameter (mm): measured in mm with a digital caliper sensitive to 0.01 mm from the harvested part of the lettuce. Root length (cm): measured from the starting point of the root to the end of the longest root using a meter. Root fresh and dry weights (g): Roots were removed from the growing medium and washed under running water to remove the solids. The roots were placed in a tared box and their wet weights were determined using a precision balance. After drying in an oven at 65 °C for 48 h, the dry weight of the roots was calculated as a percentage. Dry matter content (%): Harvested lettuce heads were weighed immediately, and fresh weight was recorded. Lettuce leaves and roots brought to the laboratory were dried in an oven at 65 °C for 48 h, and dry weights were recorded. The dry matter content (%) was calculated from the fresh and dry weights of the plants.

#### Measurements of yield and head quality

Head weight (g): Each harvested plant head was weighed on a balance with a precision of 10 g. Number of marketable leaves (number plant^−1^): The number of marketable leaves was counted after removing the non-marketable leaves from the harvested plants. Number of discarded leaves (number plant^−1^): The number of leaves with no marketing value was determined. Non-marketable leaf weight (g): Discarded leaves were weighed on a precision balance. Yield (g m^−2^): After all heads were harvested from each treatment, yield was calculated as the average head weight obtained per m^2^. Color measurement: L*, a*, b* were measured with a colorimeter (Minolta CR-400, Japan) at different points on the upper surface of the outer leaves of lettuce plants. L determines the lightness or darkness of the color from black (0 to white 100), and a and b specify the color in a color plane perpendicular to L. In the middle of the axis, the color (a = 0, b = 0) is colorless (gray-achromatic). On the horizontal axis, positive a indicates red and negative a indicates green; on the vertical axis, positive b indicates yellow and negative b indicates blue. Hue (H), which determines the basic components of color (red, yellow, blue, and green) and chroma values (C), which define the saturation and vividness of color, were obtained by calculating a and b according to the following formulas^47^.

1 $$C=\sqrt{\left({a}^{2}+{b}^{2}\right)}$$

2 $$H={\text{tan}}^{-1}\left(\frac{b}{a}\right)$$

### EC and pH of drainage solution

The EC and pH of the nutrient solution samples taken from the drainage tanks were measured twice weekly. The nutrient solution’s electrical conductivity (EC) and pH were measured with the MC-126 EC-meter and the SevenEasy pH-meter of Mettler Toledo.

### Data evaluation

Analysis of variance was applied to the data with the combined analysis of variance (ANOVA), and differences between means were determined by the LSD test with a 5% error probability by using IBM SPSS 26. The effects of treatments on plant growth and yield parameters were visualized by heatmap using the online package ClustVis (https://biit.cs.ut.ee/clustvis/). Prior to clustering, the data were standardized by unit variance scaling (z-score transformation), where each variable was centered to zero mean and scaled to unit standard deviation. For hierarchical clustering, the correlation distance similarity measure and the average linkage method were applied^48^. The data were graded using an artificial color scale with red increasing and blue values decreasing.

## Results

### Plant growth

**Fig. 3:**
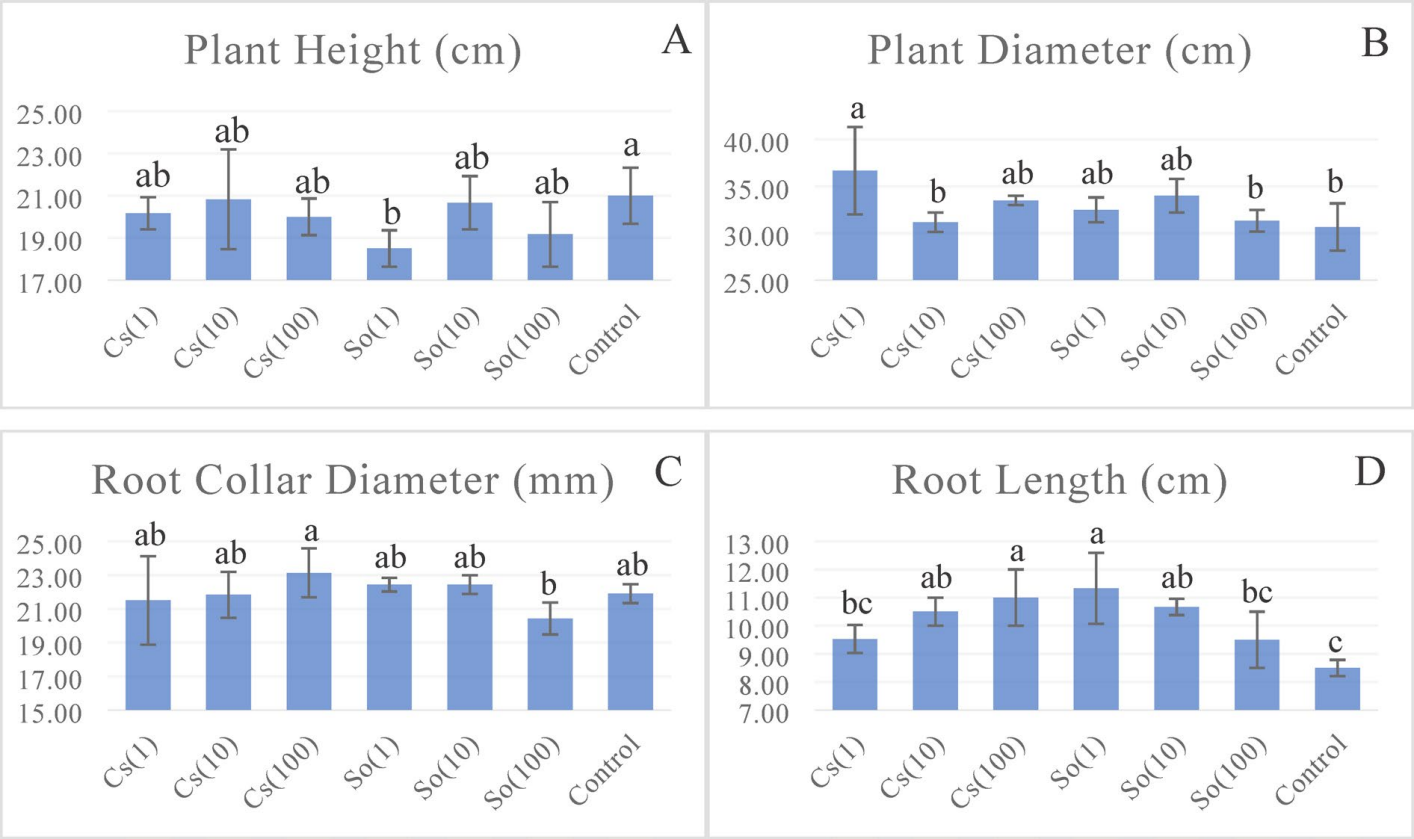
Effects of treatments on lettuce plant height* (**A**), plant diameter* (**B**), root collar diameter* (**C**), and root length** (**D**) (Statistical significance: * *P* < 0.05; ** *P* < 0.01; ns = not significant). Bars represent mean ± SD (*n* = 3). Cs(1): 0.775 g L^−1^ *Chlorella* sp; Cs(10): 0.0775 g L^−1^ *Chlorella* sp; Cs(100): 0.00775 g L^−1^ *Chlorella* sp; So(1): 0.775 g L^−1^ *S. obliquus;* So(10): 0.0775 g L^−1^ *S. obliquus;* So(100): 0.00775 g L^−1^ *S. obliquus.*

The effect of treatments on plant height was found to be statistically significant. Plant height varied between 18.50 cm and 21.00 cm (Fig. 3). In the experiment, the plant height was the highest among lettuce plants in the control group without microalgae. The height of lettuce plants in the control group was 0.8% higher than the Cs(10) treatment, 1.6% higher than the So(10) treatment, 4.1% higher than the Cs(1) treatment, 5% higher than the Cs(100) treatment, 9.5% higher than the So(100) treatment, and 12.5% higher than the So(1) treatment. The undiluted So(1) treatment obtained the lowest plant height.

The Cs(1) treatment produced the highest plant diameter of 36.67 cm. The lowest plant diameter was 30.67 cm in the control treatment. The plant diameter of lettuce plants treated with Cs(1) was 19.5% higher than that of the control treatment. The highest root collar diameter was observed in the Cs(100) treatment, followed by the So(1) and So(10) treatments, 22.43 mm. The root collar diameter of the control group was higher than Cs(1) and Cs(10) treatments, and the lowest value was observed in the So(100) treatment.

The root length of lettuce plants treated with *S. obliquus* and *Chlorella* sp. microalgae was higher than that of the control treatment. The longest root (11.33 cm) was in the So(100) treatment, while the shortest was 8.50 cm in the control treatment (Fig. 3).

**Fig. 4:**
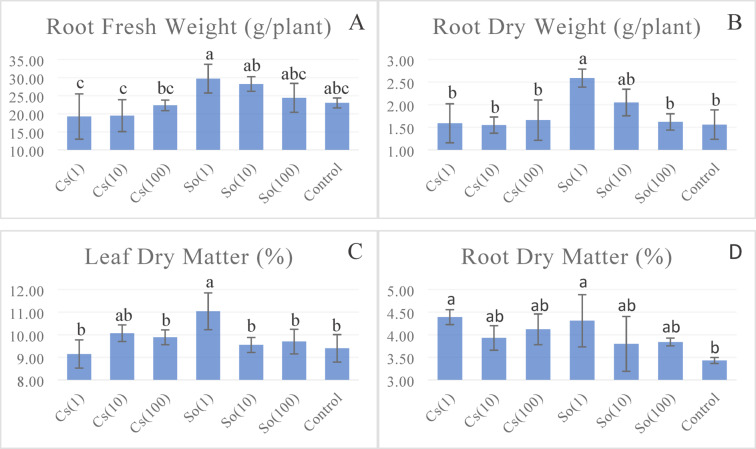
Effects of treatments on lettuce root fresh weight* (**A**), root dry weight** (**B**), leaf dry matter* (**C**), and root dry matter* (**D**) (Statistical significance: * *P* < 0.05; ** *P* < 0.01; ns = not significant). Bars represent mean ± SD (*n* = 3). ( Cs(1): 0.775 g L^−1^ *Chlorella* sp; Cs(10): 0.0775 g L^−1^ *Chlorella* sp; Cs(100): 0.00775 g L^−1^ *Chlorella* sp; So(1): 0.775 g L^−1^ *S. obliquus;* So(10): 0.0775 g L^−1^ *S. obliquus;* So(100): 0.00775 g L^−1^ *S. obliquus.*

Figure 4 displays the averages of various treatments and the statistical differences in root fresh weights. The highest root fresh weight recorded was 29.73 g plant^−1^ in the So(1) treatment, while the lowest was 19.23 g plant^−1^ in the Cs(1) treatment. When assessing the effect of different treatments on the root fresh weight of lettuce, greater root fresh weights were observed in undiluted microalgae treatments. In the So(1) treatment, root fresh weights were 29.2% and 54.6% higher than the control and Cs(1) treatments, respectively. Lettuce plants treated with Cs(1) had 19.6% lower root fresh weight than the control treatment (Fig. 4).

When root dry weight was analyzed, the highest value was obtained from the So(1) treatment, 2.59 g plant^−1^. This was followed by the So(10) treatment, 2.05 g plant^−1^. No statistical difference was observed when the other treatments were examined (Fig. 4).

Leaf dry matter content varied between 9.15 and 11.04% (Fig. 4). In the experiment, except for the So(1) and Cs(10) treatments, the other treatments were in the same statistical group regarding dry matter formation in the upper part.

Root dry matter formation rates varied between 3.43 and 4.39%. As shown in Fig. 4, S. *obliquus* and *Chlorella* sp., containing microalgae at the same dose, produced higher dry matter than the treatments without microalgae. Regarding root dry matter ratio, the Cs(1) and So(1) treatments had the highest rates of 4.39% and 4.31%, respectively. Cs(10), S(10), Cs (100), and S(100) algae doses were found in the same group, and the lowest root dry matter rate was observed in the control treatment.

### Yield

**Fig. 5:**
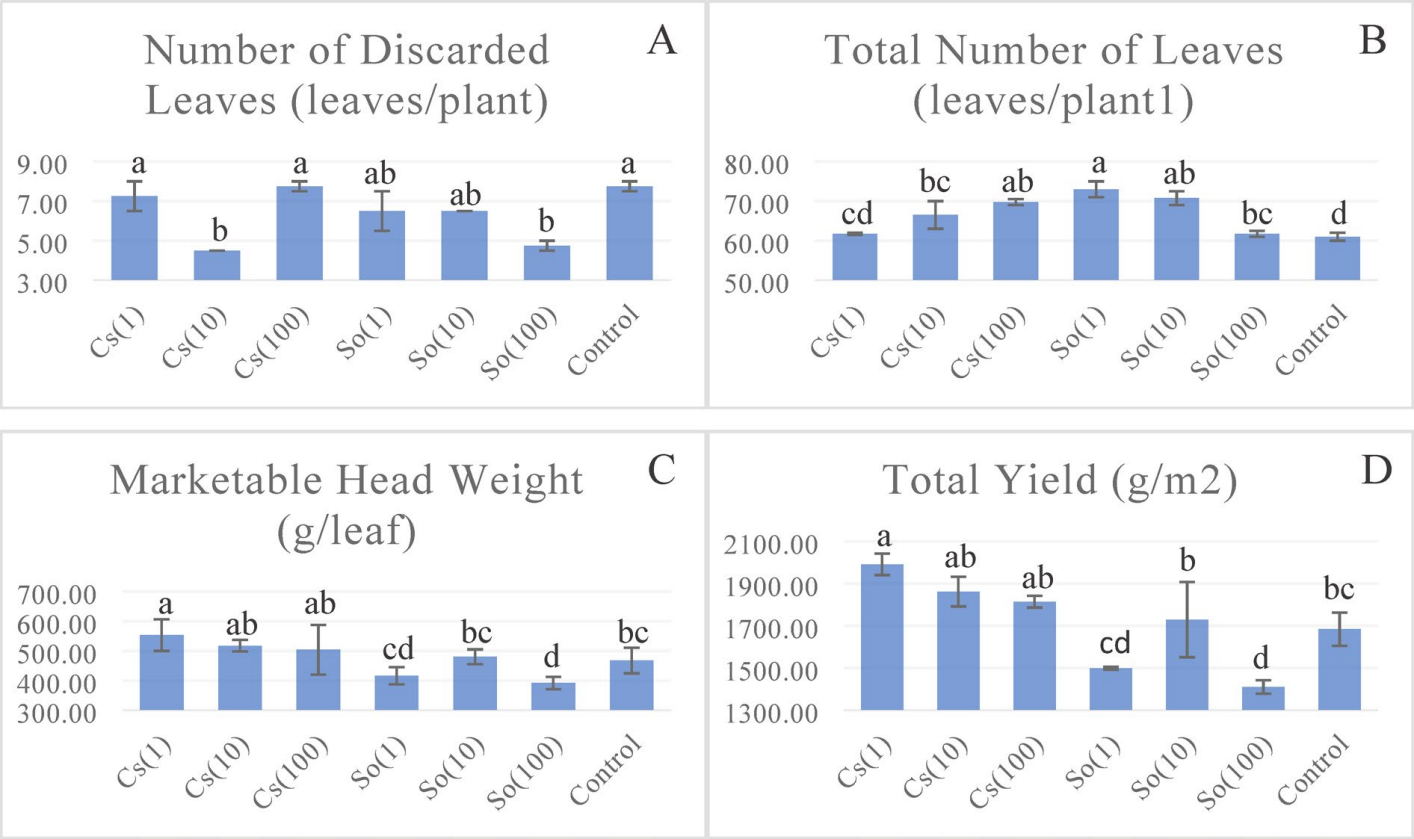
Effects of treatments on number of discarded leaves** (**A**), total number of leaves** (**B**) marketable head weight* (**C**), and total yield** (**D**) (Statistical significance: * *P* < 0.05; ** *P* < 0.01; ns = not significant). Bars represent mean ± SD (*n* = 3). Cs(1): 0.775 g L^−1^ *Chlorella* sp; Cs(10): 0.0775 g L^−1^ *Chlorella* sp; Cs(100): 0.00775 g L^−1^ *Chlorella* sp; So(1): 0.775 g L^−1^ *S. obliquus;* So(10): 0.0775 g L^−1^ *S. obliquus;* So(100): 0.00775 g L^−1^ *S. obliquus.*

When the experiment was completed and lettuce plants were harvested, all leaves on each plant were separated and counted. The number of marketable leaves of the variety was evaluated based on the application doses, and the number of leaves on lettuce was statistically affected differently by the diluted applications of two distinct microalgae species. Regarding the number of leaves, the highest number was 73 leaves plant^−1^ in the So(1) treatment, while the lowest number was 61 leaves plant-1 in the control treatment (Fig. 5). Concerning the number of unmarketable leaves, the highest numbers were observed in the control and Cs(100) treatments, while the lowest number of discarded leaves was noted in the Cs(10) treatment.

The total head weight in curly lettuce plants also refers to crop productivity in agricultural terms. The effects of different treatments on plant head weight are given in Fig. 5. The impact of treatments on plant head weight was statistically significant. The results obtained varied between 553.21 (g leaf^−1^) and 391.90 (g leaf^−1^) (Fig. 5). The total yield varied between 1991.55 (g m^−2^) and 1410.84 (g m^−2^). It was observed that the *Chlorella* sp. treatment affected the yield increase in lettuce. When the dilution doses of the *S. obliquus* treatment were examined, marketable head weight was observed to be lower than that of the control treatment.

When the effect of different treatments on the yield of lettuce was evaluated, it was determined that the yield in the Cs(1) treatment was 18.3% higher than the control treatment. The Cs(10) and Cs(100) treatments were in the same statistical group. Compared to the control, 12.4% less yield was obtained in the *S. obliquus* (1) application, and 19.4% less yield was obtained in the So(100) application. In the So(10) application, 2.7% more efficiency was obtained compared to the control application.

### Leaf color

**Fig. 6:**
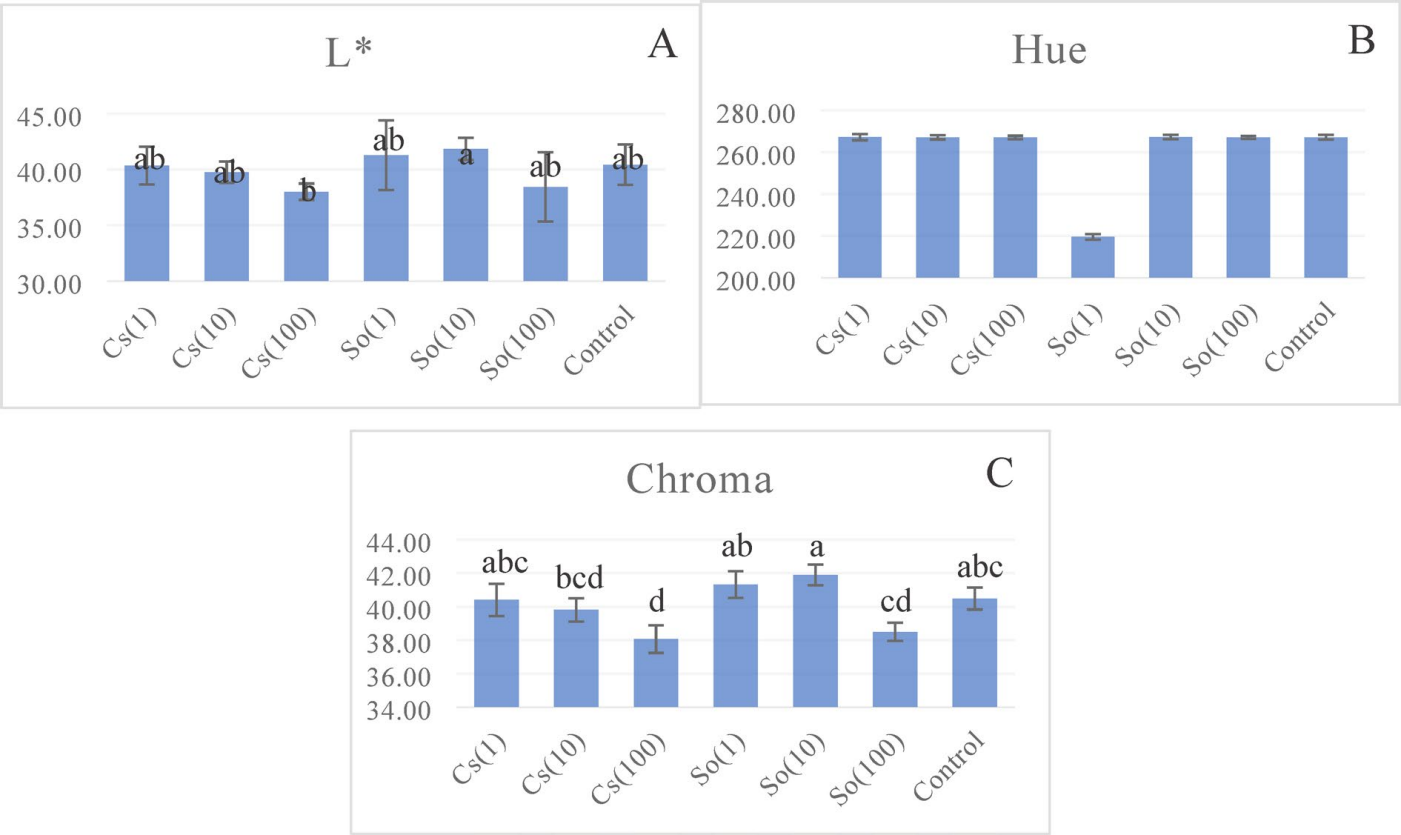
Effects of treatments on L* (**A**), Hue^ns^ (**B**), and Chroma** (**C**) (Statistical significance: * *P* < 0.05; ** *P* < 0.01; ns = not significant). Bars represent mean ± SD (*n* = 3). Cs(1): 0.775 g L^−1^ *Chlorella* sp; Cs(10): 0.0775 g L^−1^ *Chlorella* sp; Cs(100): 0.00775 g L^−1^ *Chlorella* sp; So(1): 0.775 g L^−1^ *S. obliquus;* So(10): 0.0775 g L^−1^ *S. obliquus;* So(100): 0.00775 g L^−1^ *S. obliquus.*

Leaf color is one of the most important quality criteria of vegetable species whose leaves are consumed. Depending on the species and variety, it aims to give the leaves a vivid and generally dark green color. The effect of microalgae treatments was statistically significant regarding color parameters L* and chroma. Different L* values were obtained according to the application doses, expressing leaf brightness. The highest value as 41.27 was in the So(10) treatment, while the lowest (38.44) was noted in the So(100) application. There was no statistical difference between different nutrient doses on Hue (H), which indicates color quality. When the chroma, expressing color saturation, was examined, the highest value appeared in the So(10) treatment, while the lowest was found in the Cs(100) treatment (Fig. 6).

### Nutrient solution and EC and pH of drained solution

**Fig. 7:**
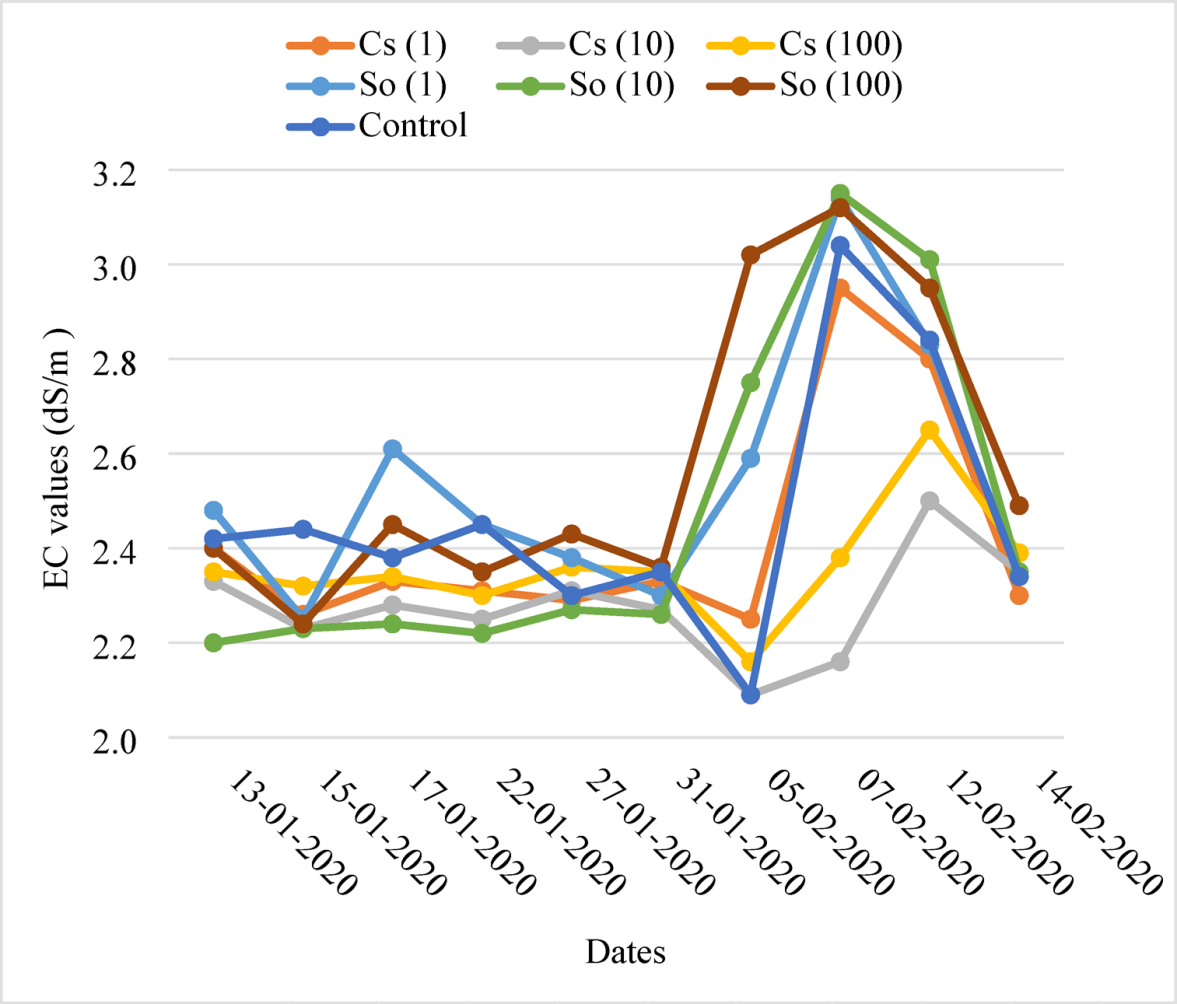
Changes in EC in the drainage solution.

**Fig. 8:**
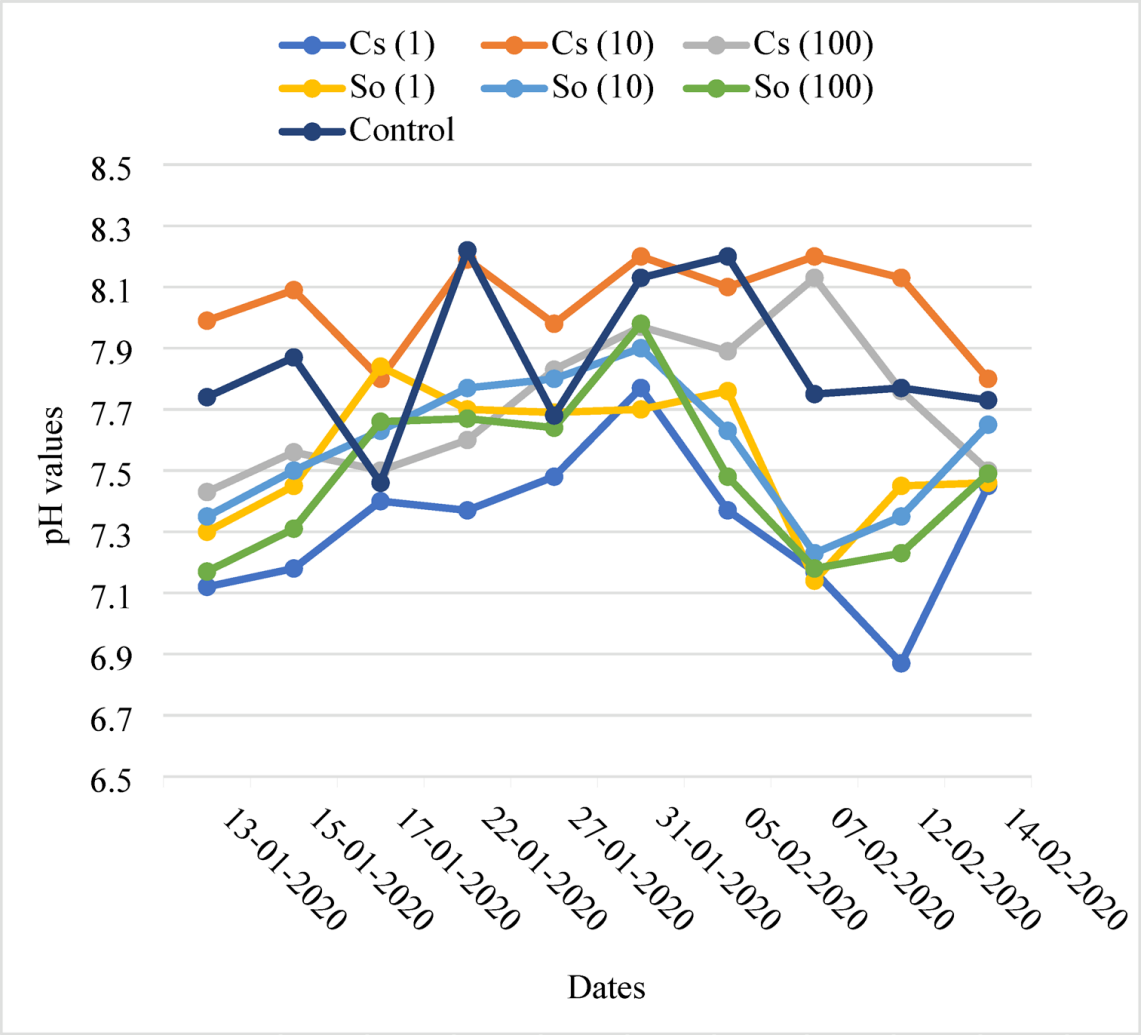
Changes of pH in the drainage solution.

Figures 7 and 8 show the EC and pH levels of drainage solutions used during the period. The EC of the nutrient solution ranged from 2.05 dS m^−1^ to 3.15 dS m^−1^, while the pH levels ranged from 6.47 to 8.22. The EC of the drainage solutions was maintained between 2 and 2.5. However, only once, on January 31, 2020, water was applied to decrease the rising EC levels due to the reduction in the amount of applied water. When the pH increased, nitric acid was used to lower it.

The EC of the nutrient solution ranged between 2.21 and 2.95 dS/m in Cs(1), 2.05–2.50 dS m^−1^ in Cs(10), 2.12–2.65 dS m^−1^ in Cs(100), 2.21–3.14 dS m^−1^ in So(1), 2.20–3.15 dS m^−1^ in So(10), 2.20–3.12 dS m^−1^ in So(100), and 2.02–3.02 dS m^−1^ in the control group. The pH ranged between 6.47 and 7.77 in Cs(1), 7.30–8.20 in Cs(10), 6.71–8.13 in Cs(100), 6.75–7.84 in So(1), 6.59–7.90 in So(10), 6.72–7.92 in So(100), and 7.20–8.22 in the control group.

### Heatmap

**Fig. 9:**
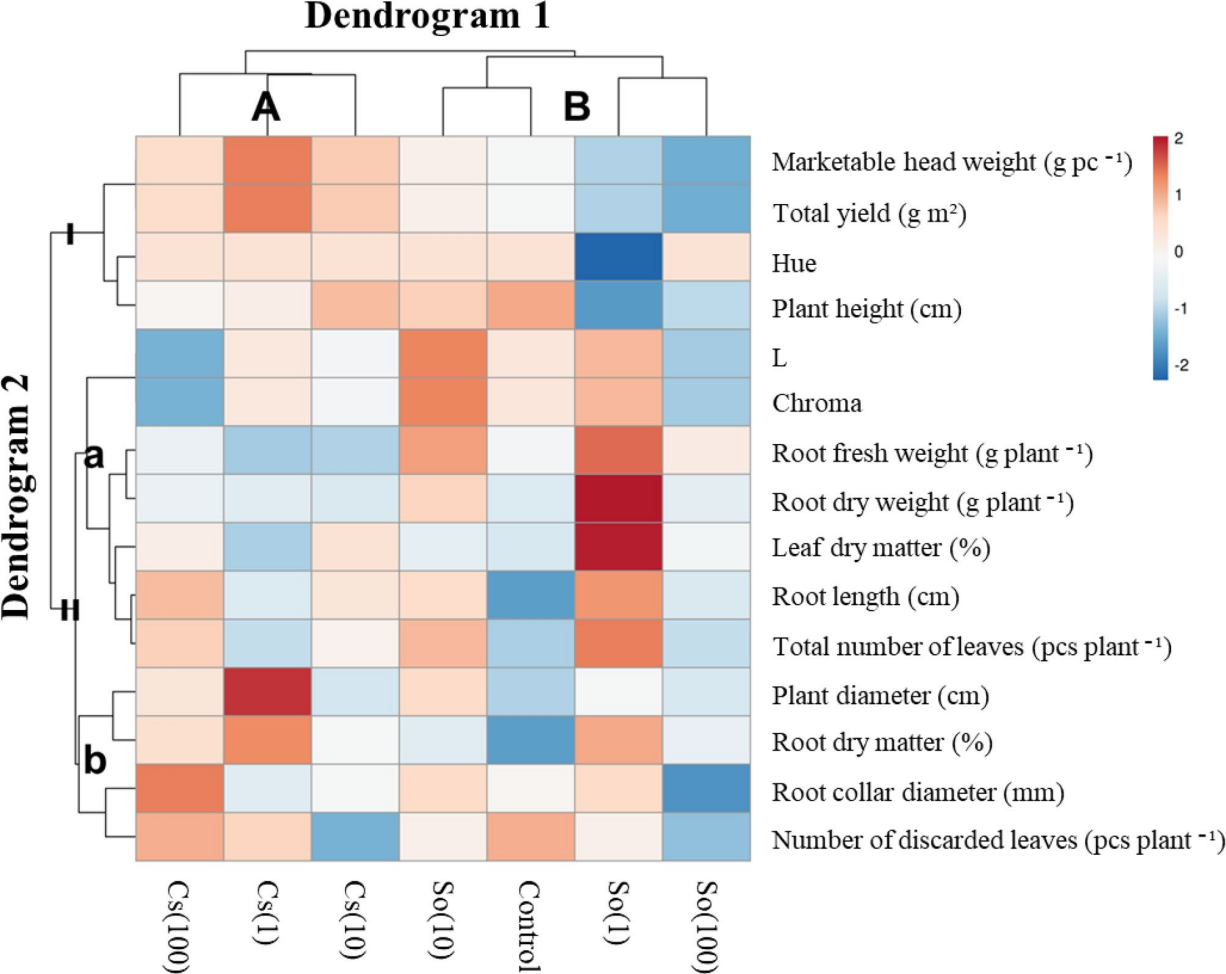
Heatmap analysis summarizing the effects of the treatments.

Heatmap analysis was applied to visualize the effects of different treatments on key growth and yield traits in lettuce cultivation (Fig. 9). Treatments (Cs(1), Cs(10), Cs(100), So(1), So(10), So(100), and Control) are displayed on the horizontal axis. Measured traits (marketable head weight, total yield, color parameters L, Hue, Chroma, plant height, plant diameter, root length, root collar diameter, root fresh and dry weight, root and leaf dry matter, number of discarded and total leaves) are listed on the vertical axis. The data were standardized using unit variance scaling, and hierarchical clustering was performed with correlation distance and average linkage. The resulting dendrograms clearly separated treatments into two main groups (clusters A and B) and traits into three groups (clusters I, II-a, and II-b). This clustering highlights how certain microalgae treatments grouped and were associated with improved yield and quality parameters, while others clustered closer to the control. The color gradient, with red indicating values above and blue below the mean, further illustrates these treatment–trait relationships and provides an integrative view of the overall plant response. These patterns support our hypothesis that specific microalgae strains and dosages differentially influence lettuce performance. Of the two dendrograms resulting from the heatmap analysis, Dendrogram 1 showed the trial subjects, and Dendrogram 2 showed the parameters affecting the distribution. Two main groups were formed in Dendrogram 1, the cluster on the left (A) included the Cs treatments, and the cluster on the right (B) included the So and control treatments. The Cs(100) subject under Dendrogram 1 was divided into groups as it affected the parameters of root collar diameter and the number of leaves discarded: Cs(1) marketable head weight, the total yield, plant diameter, and root dry matter; Cs(10) the number of leaves discarded; So(10) L and chroma; the control plant height and the number of leaves discarded; So(1) Hue, root fresh weight, root dry weight, leaf dry matter, root length, and the total number of leaves; and So(100) marketable head weight, total yield and root collar diameter. In Dendrogram 2, the measured parameters were grouped to reveal the effects of the experimental subjects. In the first panicle of Dendrogram 2 (I), marketable head weight and total yield, which produced high figures in Cs(1), formed one sub-panicle, while Hue and plant height parameters made up the second sub-panicle. In the other panicle (II), biomass, color, and leaf number data were created for sub-panicles under the second panicle. When the sub-clusters were examined individually, II-a combined the color, root weights and lengths, leaf dry matter, and the total number of leaves, excluding Hue. In cluster II-b, plant and root collar diameter, plant dry matter, and number of discarded leaves were grouped.

## Discussion

Microalgae are increasingly recognized for their potential in sustainable agriculture. They could provide nutrients and produce molecules stimulating plant growth^49^. The correct formulation and/or dosing is crucial to increasing efficiency.

In our research, we hypothesized that live freshwater microalgae in substrate culture could enhance the growth and yield of lettuce plants. We compared two microalgae species (*S. obliquus* and *Chlorella* sp.) and aimed to determine the optimal dosage. The treatments significantly affected all measured parameters, and in most cases, microalgae application had a positive impact depending on strain and biomass density, except for plant height. Plant height and diameter may respond differently because environmental factors^50^, cultivation practices, and management strongly influence height^51^. In our study, similarly to Miao et al. (2023), light intensity seems to have a more pronounced effect on plant height. However, inoculant formulation can also affect the success^52^, which could contribute to our result. Contrary to plant height, plant diameter increased compared to the control.

The root collar diameter and root length of lettuce plants treated with *S. obliquus* and *Chlorella* sp. were higher than those treated with the control treatment. However, strain densities affected both parameters. Higher values were obtained in higher densities of *Chlorella* sp., while in lower densities of *S. obliquus.* This trend was also determined in root biomass. Regarding marketable head weight and total yield, *Chlorella* sp. performed better, and the undiluted dose (0.775 g fresh weight L^−1^) was the most effective.

In previous studies, Aydöner and Daşgan^53^ reported that the addition of *Chlorella vulgaris* to complete dose nutrient solution and full and reduced nutrient solutions did not affect plant height, the number of leaves, the number of clusters, stem and leaf fresh weight, and leaf area in greenhouse tomato cultivation. In a study evaluating the potential of *Chlorella vulgaris* and *Tetradesmus obliquus* as microalgae as biostimulant sources under in vivo conditions, the effects of *Chlorella vulgaris* were examined on morphological parameters such as leaf length, leaf number, fresh and dry weight of leaves, leaf surface area, and leaf chlorophyll content of lettuce seedlings. According to the results of this study, the effect of *T. obliquus* showed no difference compared to the control, while *C. vulgaris* positively affected lettuce growth, primarily by increasing leaf length, dry weight of leaves, and leaf surface area^54^. *C. vulgaris* and *S. obliquus* grown in agricultural drainage from maize cultivation did not show a significant difference in lettuce production and increased the lettuce biomass^55^. However, in our heatmap analysis, two main groups were formed in Dendrogram 1 for each species, and showed different performance. Application concentration requires optimization to stimulate phytohormones and nutrient supplementation^56,57^.

Microbial inoculants are affected by the root exudates, environmental conditions^58^, physicochemical properties of the soil^59^, and/or formulations^60^. In soilless culture, the EC level of substrate, which affects solubility and accessibility of essential nutrient elements, is of great importance for microalgae metabolism, particularly lipid accumulation of *Chlorella* sp., which could be related to the reduction^61^. Our results showed that the measured parameters varied according to species and strain densities, which could be related to the temperature^62^ and particularly with characteristics of the rhizosphere environment such as nutrient availability, pH ^63^, and EC ^61^. The increased performance could be related to hormone-like substances^64^. Santoro et al. ^65^ indicated that the transcriptomic response of lettuce seedlings clearly shows that *C. vulgaris* activates both the auxin biosynthesis and transduction pathways, whereas *S. quadricauda* upregulates the cytokinin biosynthesis pathway, most probably due to the differences in the amount of beneficial components.

Microalgae show significant potential for biofuel production in renewable energy, biopharmaceutical, and nutraceutical sectors due to their rapid growth, high biochemical versatility, and CO_2_ sequestration capacity^66,67^. Key determinants include biomass accumulation, lipid productivity, and fatty acid composition, which collectively determine biofuel quality and yield^68^. Microalgae can theoretically convert 9–10% of solar energy into biomass, achieving maximum productivity of ~ 77 g m^−2^ day^−1^. However, photobioreactor applications yield significantly lower biomass due to suboptimal light distribution, shading effects, and photosynthetically active radiation losses^67^. In agriculture, microalgae may serve as more efficient and economical biostimulants, particularly when utilizing low-cost resources.

This study aimed to evaluate liquid microalgal biomass as a biostimulant/biofertilizer without separating the biomass from the liquid medium. This direct application approach offers significant commercial adtantages by eliminating energy-intensive harvesting processes and reducing machinery costs. Additionally, plant nutrients not fully consumed by microalgae remain in the medium, creating and enriched biostimulant/biofertilizer with enhanced nutritional value for crop applications.

However, the application of liquid microalgal biomass as a biostimulant or biofertilizer should be considered within the broader framework of agronomic and cultural practices^69^, rather than as an isolated measure. Gruda et al.^69,70^ also recommend combining biostimulants with biofortification strategies to enhance the nutritional quality of greenhouse vegetables further. In addition, the choice of genetic material, including cultivar selection^71^, plays a decisive role, as do prevailing climatic conditions, which strongly influence both yield and quality^72^. Taken together, these factors highlight the need for integrated approaches in greenhouse production, where microalgal applications are aligned with cultivar choice, biofortification, and environmental management^69,70^, to achieve optimal and sustainable outcomes for lettuce and other vegetable crops. Last but not least, to achieve high yield together with high quality, a compromise—what Gruda et al. describe as the “golden mean”—should be sought^73^.

## Conclusion

This study effectively illustrated how applying live microalgae can improve lettuce yield and growth in soilless greenhouse systems. Our hypothesis that live microalgae cultures would enhance plant performance was validated by the results. In particular, the most successful treatment was *Chlorella* sp. applied at an undiluted dose (0.775 g fresh weight L^−1^), which greatly increased the weight of the lettuce heads and the overall yield. Although *S. obliquus* also had positive effects, it performed best at a concentration that was ten times diluted (0.775 g fresh weight L^− 1^). This result validated that there are specific optimal concentrations for optimizing lettuce production with each microorganism and supported the hypothesis that microalgae species differ in their efficacy.

These results highlight the usefulness of incorporating particular live microalgae species into soilless cultivation in controlled environments. The capacity of *Chlorella* sp. to significantly boost lettuce yield at its optimal concentration offers a viable and sustainable method of improving greenhouse production efficiency. The precise underlying mechanisms causing these beneficial plant-microalgae interactions should now be clarified in future studies. Additionally, application strategies for a variety of crops and cultivation conditions should be further optimized, and the long-term sustainability and economic feasibility of integrating these microalgae-based biostimulants into commercial soilless systems should be assessed.

## Data Availability

Data will be made available from the corresponding author upon request.
